# Radiation sterilisation of cultured human brain tumour cells for clinical immune tumour therapy

**DOI:** 10.1038/sj.bjc.6601467

**Published:** 2004-01-06

**Authors:** C Bauréus-Koch, G Nyberg, B Widegren, L G Salford, B R R Persson

**Affiliations:** 1 Department of Radiation Physics, Lund University Hospital, Klinikgatan 7, SE 221 85 Lund, Sweden; 2 Tumour Immunology, Lund University Box 7031, SE 22007, Lund, Sweden; 3 Department of Neurosurgery, Lund University Hospital, SE 221 85 Lund, Sweden; 4 The Rausing Laboratory, Lund University, S-221 85 Lund, Sweden

**Keywords:** glioblastoma multiforme, ^137^Cs-*γ* rays, mean lethal dose, MTT, ^3^H-thymidine, interferon-*γ*, immunisation

## Abstract

The aim is to investigate the radiosensitivity of noninfected cultured human glioma cells to ascertain that intracutaneously administered cells are viable enough to produce interferon-*γ* but not able to proliferate. Cell cultures were established from five patients undergoing brain tumour surgery. By karyotyping, we found four malignant (three glioblastoma multiforme (GBM), one giant cell glioma) and one normal. The cells were irradiated with ^137^Cs-*γ* rays at absorbed dose levels of 0, 20, 40, 60, 80, 100 and 120 Gy. The fraction of viable cells was examined by MTT incorporation assay. The average of the data obtained from three GBM cell cultures was fitted to an exponential model. The parameters were: extrapolation number *n*=0.85±0.10, mean lethal dose *D*_0_=12.4±3.2 Gy and an additional uncertainty parameter *δS*=0.14±0.03. By setting *δS*=0, the corresponding values of the parameters were *n*=0.86±0.16 and *D*_0_=30.0±8.1 Gy. The rate of proliferation was examined by ^3^H-thymidine incorporation. The average of the proliferation data obtained from three GBM cell cultures was fitted to an exponential model yielding *n*=0.943±0.005 and *D*_0_=5.8±0.5 Gy for *δS*=0.057±0.005, and by setting *δS*=0, *n*=1.00±0.02 and *D*_0_=8.4±1.6 Gy. No outgrowth of plated cells was observed after 4 weeks at an absorbed dose of 100 Gy. This absorbed dose is recommended for irradiation of 2 × 10^6^ glioma cells used for clinical immunisation.

The most malignant type of brain tumours, glioblastoma multiforme (GBM)– or astrocytoma grade IV (according to the WHO classification) as well as giant cell gliomas (GCGs) are among the most therapy-resistant human cancers (Salford *et al*, 1988). The tumour front of this tumour, consisting of highly proliferating cells, grows by progressively killing the surrounding normal brain cells, neuronal and glial. In autopsies, glioma cells, single or in small clusters, are demonstrated in the whole brain, including the brain stem of patients with subcortical tumours (Burger *et al*, 1988). Radiotherapy and chemotherapy have hitherto proven not to be efficient enough for cure (Sheline, 1977; Stenning *et al*, 1987).

We believe that the key for success in the treatment of GBM would be a method to reach the migrating glioma cells, also called ‘guerrilla cells’. Such an efficient treatment regime could be ‘immunogenic tumour therapy’. In a brain tumour rat model, >40% of the rats survived >30 weeks after immunisation with syngeneic brain tumour cells transfected with the rat interferon-*γ* (IFN-*γ*) gene. The cells were injected subcutaneously 1 to 3 days after the inoculation of nontransfected glioma cells in the brain of the rats. If not treated with immunisations, these animals develop lethal intracerebral gliomas within 3 to 4 weeks (Visse *et al*, 1999).

In the translation of this model to the human situation, it is of great importance for safety and efficiency to know the radio sensitivity and survival characteristics of the cultivated cells. We have investigated the efficiency of sterilisation of human malignant brain tumour cells in preparation for the clinical research programme *Brain ImmunoGene Tumour Therapy* (‘BRIGTT’) approved by the Medical Products Agency in Sweden (Salford *et al*, 2002). The immunisation will take place by administration of radiation-sterilised autologous tumour cells that have been genetically engineered to produce human IFN-*γ* and green fluorescent protein as immune enhancers, in the dermis of the upper arm. It is important that the radiation dose used for sterilisation of these cells will inhibit the proliferation of the genetically modified tumour cells but not block the metabolic activity and the production of recombinant proteins.

Previous studies of the intrinsic cellular radiation sensitivity of noninfected human glioblastoma cell lines have used low radiation dose levels in order to investigate the shoulder of the survival curves (Malaise *et al*, 1986; Taghian *et al*, 1992). In the present study, however, we examine the radiation survival characteristics at much higher radiation dose levels. Thus, our aim is to investigate the radiation characteristics of noninfected cultured human glioma cells to ascertain that intracutaneous administered cells are viable enough to produce IFN-*γ* but not able to proliferate. In this study, we also included an apparently normal brain cell culture (as monitored by karyotyping) from a human GBM specimen in order to give some information on the effect of irradiation in normal brain cells. In theory, normal cells can induce autoimmune reactions and should not be present when genetically modified cells are injected in the patients.

## MATERIALS AND METHODS

### Media

Cells were cultured in complete Iscove's modified Dulbecco's medium (IMDM) medium (Life Technology, Paisley, UK) supplemented with 15–20% foetal calf serum (FCS) (Sigma Chemical Co., St Louis, MO, USA), penicillin (Labora, Malmoe, Sweden), streptomycin (Labora, Malmoe, Sweden), Na pyruvate (Life Technology), MEM (nonessential amino acids) (Life Technology, Paisley, UK) and *α*-thioglycerat (Sigma).

Lysis solution used in the 3-[4,5-dimethylthiazol-2-yl]-2,5-diphenyltetrazolium bromide (MTT) assay: 20% w v^−1^ of SDS was dissolved at 37°C in a solution of 50% of DMF (*N*,*N*-dimethyl formamide) and demineralised water, pH was adjusted to 4.7 by adding 2.5% of 80% acetic acid and 2.5% 1 M HCl.

### Cells

Cells were obtained from cell cultures of biopsies from five patients with malignant brain tumours – four with GBM and one with a GCG. From each patient undergoing surgical removal of the brain tumour, 100–3000 mg of tumour tissue was collected, minced and digested with an enzyme solution containing 0.5 mg ml^−1^ collagenase type VIII (Sigma) and 20 mg ml^−1^ DNAse I (Sigma) dissolved in IMDM without FCS. After 3 × 20 min of digestion at 37°C with continuous gentle agitation, the resultant cell suspension was centrifuged at 150 **g** for 15 min. The pellet was suspended with 10 ml complete IMDM medium and seeded on Primaria plastic tissue culture flasks (25 cm^2^, Labora, Malmoe, Sweden) to be kept in a humidified incubator at 37°C in 10% CO_2_.

The cells were cultivated for 2–4 months before they were karyotyped at the Department of Clinical Genetics at Lund University Hospital, Sweden. Human malignant glioma cells grow rather slowly in tissue cultures. The doubling time is between 70 and 100 h for most primary glioma cell cultures. On the other hand, normal cells in tissue culture have a doubling time of approximately 24 h. Owing to these circumstances, cultures from human brain tumour specimens are initially overgrown by normal cells, for example, endothelial cells, fibroblasts and other nonmalignant cells. After a few months, *in vitro* culture normal cells cease to divide and the malignant cells will prevail. It was possible to establish the growth of pure malignant glioma cells in more than 50% of the cultures. The cells from the fourth GBM patient had not reached a malignant stage and only cells with normal karyotype grew in this culture when they were harvested.

### Karyotyping

The five cultures were called GCG (originating from GCG patient # 1212), with the karyotype: 41–44,XX,del(2)(p22–23),del(4)(p11),del(5) (p11),+7,−10,−11,−13,−16,−17,add(22)(q13), +mar[cp5] (14 cells, all aberrant), GBM1 (originating from patient # 1141), with the karyotype 108–121, XX, add(1)(q21), del(1)(q12), add(6)(q?15), add(19)(p13), +?der(?)t(?;10)(?;q11), inc[cp4] (11 cells, all polyploid and complex), GBM2 (originating from patient # 1151), with the karyotype 69–77, XXY, +Y, +1, add(1)(p11) × 2, −4, +7, +7, +7, +8, +9, add(9)(p11) × 2, −10, +13, −14, −15, −17, −18, der(19)t(?17;19)(q21;q13), +20, +21, +22, +2mar[cp] (11 cells, all aberrant), GBM3 (originating from patient # 1160), with the karyotype 63–66, X, del(1)(q21), +?add(3)(q11), der(4;14)(q10;q10), add(11)(q23), add(12)(p11), +?14, +mar, inc[cp2]/125, X, ?del(1)(q21), add(12) (p11), inc[2] (11 cells, all aberrant) and normal cells (N1, originating from GBM patient # 1211) 14 cells, with normal karyotype.

### Irradiation

The cells were transferred from the culture flasks to 15 ml centrifuge test tubes (Nanclon, Nalge NUNC International, Denmark) and stored in a melting ice bath before irradiation with ^137^Cs-*γ* rays using a Gammacell 2000 (Mølsgaard Medical, Risø, Denmark) source at a dose rate of 4.0 Gy min^−1^. During the irradiation, the cells were kept at room temperature and the cell density was 2 × 10^4^ cells ml^−1^. The cells were kept in a serum-free medium (IMDM-0) that does not allow the cells to grow during the procedure. The cells were irradiated to 0, 20, 40, 60, 80, 100 and 120 Gy that correspond to irradiation times up to 30 min. In order to keep the cells at room temperature for equal time, all samples were kept for 30 min at room temperature before they were placed in a melting ice bath. Nonirradiated control cells were kept in 15 ml centrifuge test tubes in serum-free medium (IMDM-0) and were stored for 30 min at room temperature and on melting ice during the procedure.

### MTT assay

The viability condition of irradiated cells is obtained from studies of how the mitochondria in living cells transforms MTT to formazan salt (Denizot and Lang, 1986), which is insoluble in the medium. The salt can be dissolved by sodium dodecyl sulphate and the absorption at 570 nm is closely correlated to the number of viable cells in the sample. After irradiation, the cells were plated in flat-bottomed 96-well plates (Nanclon), 5000 cells well^−1^, and kept in a humidified incubator with 10% CO_2_ at 37°C for 5 days. Then, 25 *μ*l of MTT stock solution (5 mg ml^−1^) was added to each well, followed by incubation at 37°C and 10% CO_2_ for 2 h. After 2 h of incubation, 100 *μ*l of lysis solution was added to each well and further incubation at 37°C and 10% CO_2_ was required for at least 6 h. The optical densities at 570 nm were measured using an ELISA reader (Multiskan MS, Labsystems, Finland).

### ^3^H-thymidine incorporation in DNA

Incorporation of tritium-labelled thymidine in DNA is used to study the rate of DNA synthesis and thus the rate of the cell division (proliferation) (Yoffey *et al*, 1959, 1961; Devik, 1962; Quarteyp and Yoffey, 1968). The irradiated tumour cells were plated in flat-bottomed 96-well plates (Nanclon, Nalge NUNC International, Denmark), 5000 cells well^−1^, and kept in a humidified incubator in 10% CO_2_ at 37°C for 5 days. An activity of 0.5 *μ*Ci (18.5 kBq) ^3^H-thymidine (Amersham, UK) was thereafter added to each well, followed by incubation of the cells for 6 h in 10% CO_2_ at 37°C. The samples were harvested, using double-distilled water to lyse the cells, and the lysate was filtrated through glass fibre membranes (Wallace Oy, Finland) on which materials of high-density molecules were collected. In each assay, the membranes were dried and the radioactivity was measured in a liquid scintillation counter (Wallace Microbeta, Wallace Oy, Finland). The analysis of each sample was performed in six replicates. The recorded count rate (c.p.m.) value is a measure of the number of cells that have undergone cell division or DNA repair during the 6 h of incubation with ^3^H-thymidine.

### Plating efficiency

Approximately 1 × 10^6^ cells from GCG, GBM1, GBM2, GBM3 and N1 cultures were irradiated with an absorbed dose of 100 Gy. The irradiated cells were plated in tissue culture flasks with complete IMDM medium and were incubated at 37°C and in 10% CO_2_ for up to 4 weeks. The cells were followed by optical inspection about every second day under an inverted phase-contrast microscope during a 4-week period. In these adherent tissue cultures, nondividing cells flatten out on the surface and look greyish and dark. Actively proliferating cells are lighter and if in mitosis, they round up and appear radiant. When the cells die, they float up to the surface of the medium and disintegrate. Hence, the total number of cells in the irradiated cultures decreases over time and after about 3–4 weeks, there were very few proliferating cells to be observed.

### Cell survival model

In the present work, we are mainly interested in studying the survival of cells exposed to large absorbed doses >20 Gy. The survival of mammalian cells *in vitro* irradiated with large absorbed dose is modelled according to an exponential model (Tubiana *et al*, 1990).

In our experiments, however, we find a certain fraction of cells proliferating after having received absorbed doses of 20 Gy and above. Most models of cell survival do not take into account this fraction of cells, which are still proliferating at very high values of the absorbed dose. Therefore, we add a term *δS* to the traditional equation of survival fraction *S* as follows:


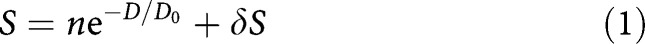


where *S* is the survival fraction, *D*_0_ is the mean lethal dose for which the survival fraction *S*=1/*e*, *D* is the administered absorbed dose, *n* is the extrapolation number at *D*=0 that might be interpreted as the number of sublethal events and *δS* is the extrapolation number at infinite absorbed dose that might be interpreted as either a methodological uncertainty number or a fraction of highly radioresistant cells.

The survival fraction of the MTT assay gives the probability or fraction of viable cells, *W*(*V*) that includes both apoptotic and proliferating cells. On the other hand, the survival ratio of the ^3^H-thymidine assay gives the fraction of cells proliferating (*L*) and thus also viable (*V*). This fraction or probability can be written as *W*(*V*∩*L*). To guarantee sterile conditions, we wish to evaluate the probability of viable cells able to proliferate after irradiation. This is equal to the conditional probability *W*(*L*∣*V*) of cell proliferation (*L*) given that the cells are viable (*V*) after the irradiation exposure (Lindgren, 1976), which is well defined only when *W*(*V*)>0:


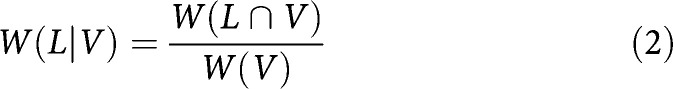


where *W*(*L*∣*V*) is the probability of proliferation of cells still viable after radiation, *W*(*L*∩*V*) is the probability of cells to proliferate and being viable, that is, *S*(^3^H-thymidine) and *W*(*V*) is the probability of cells being viable, that is, *S*(MTT).

The absorbed dose that corresponds to a certain probability, *W*(*L*∣*V*), of proliferation of cells still viable after radiation equals the exponential term in equation (1) that can be expressed as follows:


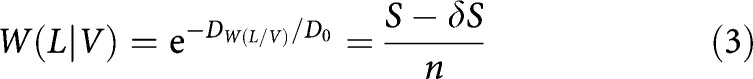


Thus the absorbed dose, *D*_*W*(*L*∣*V*)_, which corresponds to a certain probability, *W*(*L*∣*V*) becomes


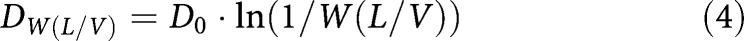


From this equation the absorbed dose for sterile condition, that is, *W*(*L*∣*V*)=10^−6^ (*w*=E−6) can be given as:


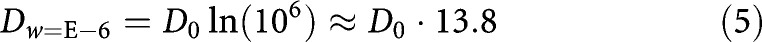


## RESULTS

Cells from four malignant brain tumour cultures (GCG, GBM1, GBM2 and GBM3, respectively) and one from a normal brain cell culture (N1) were irradiated at absorbed doses of 20, 40, 60, 80, 100 and 120 Gy at a dose rate of 4.0 Gy min^−1^. After irradiation, the cells were plated in 96-well plates (5000 cells well^−1^) and evaluated either by incorporation of ^3^H-thymidine or by an MTT assay at 5 days after irradiation.

### ^3^H-thymidine incorporation assay

**Figure 1:**
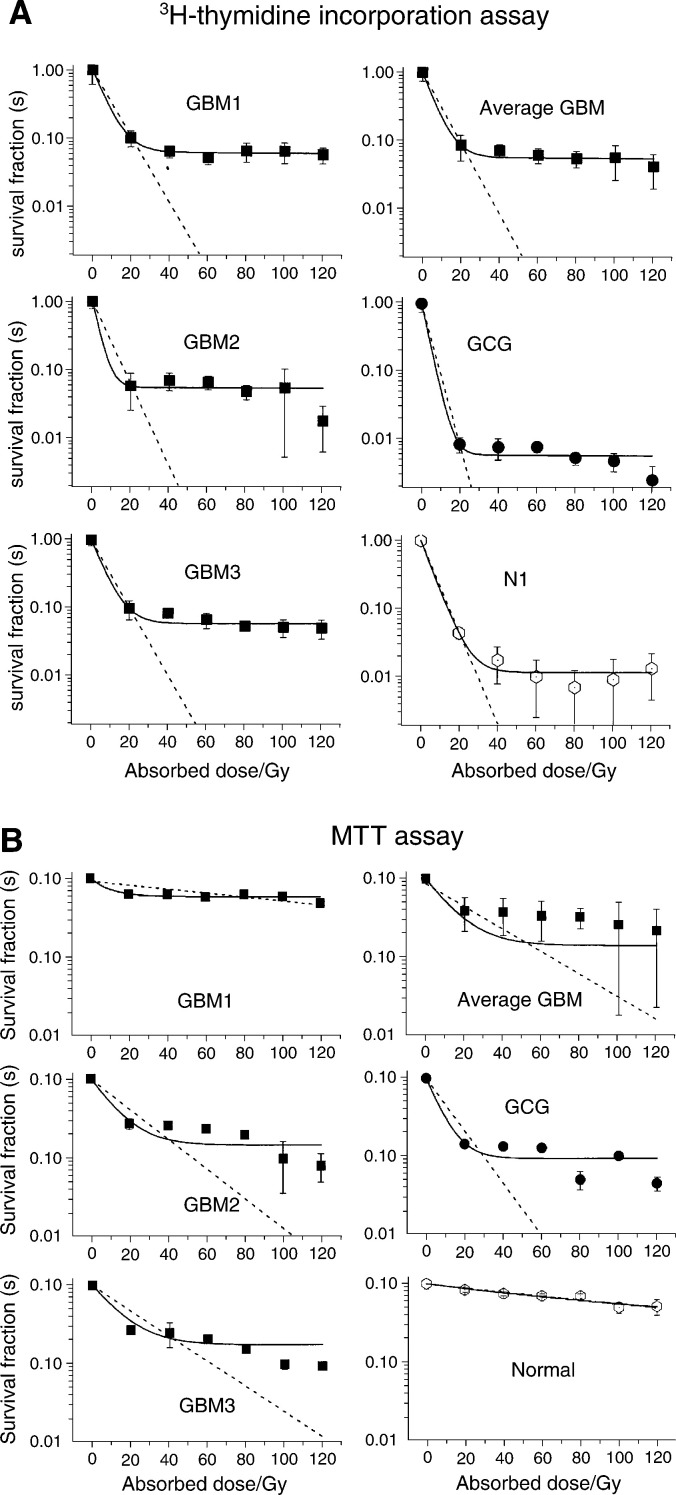
(**A**) Surviving ratio from ^3^H-thymidine incorporation as a function of absorbed dose for the irradiated cells of patients GM 1, GM 2, GM 3, GC 1 (of malignant karyotype) and N1 (of normal karyotype). (**B**) Surviving fractions from MTT assays as a function of absorbed dose for the irradiated cells from patients GMB 1, GMB 2, GMB 3, GC (of malignant karyotype) and of normal karyotype. Keys to the abbreviations are given under the paragraph ‘Karyotyping’.

The cell division examined by the ^3^H-thymidine incorporation assay is given as the count rate of ^3^H plotted as a function of the absorbed dose. The surviving ratios from the ^3^H-thymidine incorporation for the different cell cultures are plotted against the absorbed dose in Figure 1a

for the individual cell lines. The surviving ratio of normal cells and the average of GBM cell lines are displayed separately in the same figure. The incorporation of ^3^H-thymidine reflects both the unscheduled DNA synthesis (DNA repair) as well as DNA synthesis reflecting cells entering the S phase. The incorporation of ^3^H-thymidine is measured by a ‘6 h pulse labelling’ 5 days after the irradiation. In the cell survival experiments, we observed a dramatic reduction in the number of cells about 1 week after irradiation. The results obtained by fitting the data of ^3^H-thymidine incorporation assay to the exponential model for cell survival (equation (1)) are given in Table 1

**Table 1:** Radiation sensitivity parameters *n*, *D*_0_ and *δS* for irradiated cells obtained from fitting ^3^H-thymidine incorporation assay data of cells from patients GBM1, GBM2, GBM3, GCG (malignant karyotyped) and N1 (normal karyotyped)

**Uncertainty factor**	***δS*=0**		***δS*≠0**		
**Fitting parameters**	***n***	***D*_0_ (Gy)**	***δS***	***n***	***D*_0_ (Gy)**
*Cell lines*					
GBM1	1.000±0.013	8.98±1.78	0.063±0.002	0.937±0.003	6.29±0.25
GBM2	1.000±0.017	7.21±1.90	0.054±0.007	0.946±0.009	3.39±3.35
GBM3	1.000±0.015	8.79±1.37	0.060±0.005	0.940±0.006	8.79±1.37
Average GBM1–3	1.000±0.016	8.38±1.62	0.057±0.005	0.943±0.005	5.76±0.50
GCG	1.000±0.001	4.22±0.55	0.006±0.001	0.994±0.001	3.36±0.48
N1(Normal)	1.000±0.003	6.47±0.54	0.012±0.002	0.998±0.002	5.86±0.22

The average GM values are calculated from patients GBM1, GBM2 and GBM3.

.

### MTT assay

The viability, that is, surviving fractions derived from the recorded optical densities of the MTT assay, of the different cell cultures, are plotted against the dose in Figure 1b. In Figure 1b the surviving fraction values of normal (N1) and the four malignant cell cultures, GBM1, GBM2, GBM3 and GCG are given for different doses between 0 and 120 Gy in 20 Gy increments. The figure shows that for all these cell cultures, there is a decreasing fraction of viable cells with increased absorbed dose. In Figure 1b, we have also plotted the average surviving fraction of all the GBM tumour cell cultures, whereas the normal cell culture is displayed separately. The measurement is recorded at 5 days after irradiation and at this time, a certain number of cells are no longer viable, for example, detectable as physiological functional cells. Since the amount of viable cells is correlated to the absorption of the dissolved formazan salt in the MTT assay, the optical density plotted in Figure 1 gives the viability as a function of the absorbed dose. The results obtained by fitting the data of the MTT assay to the exponential model for cell survival (equation (1)) are given in Table 2

**Table 2:** Radiation sensitivity parameters *n*, *D*_0_ and *δS* for irradiated cells obtained from fitting of the MTT assay data of cells from patients GBM1, GBM2, GBM3, GCG (malignant karyotyped) and N1 (normal karyotyped)

**Uncertainty factor**	***δS*=0**		***δS*≠0**		
**Fitting parameters**	***n***	***D*_0_ (Gy)**	***δS***	***n***	***D*_0_ (Gy)**
*Cell lines*					
GBM1	0.92±0.07	171±45	0.59±0.023	0.41±0.04	9.6±5.0
GBM2	0.98±0.08	22.8±5.9	0.14±0.04	0.86±0.06	11.6±3.2
GBM3	0.99±0.052	26.9±3.6	0.17±0.04	0.83±0.05	13.2±4.1
Average GBM1–3	0.86±0.16	30.0±8.1	0.14±0.03	0.85±0.10	12.4±3.2
GCG	1.00±0.04	13.0±3.4	0.10±0.02	0.91±0.03	7.1±2.2
N1(Normal)	0.99±0.03	166±14	0.24±0.31	0.75±0.30	108±75

The average GBM values are calculated from patients GBM1, GBM2 and GBM3.

.

### Plating efficiency

**Figure 2:**
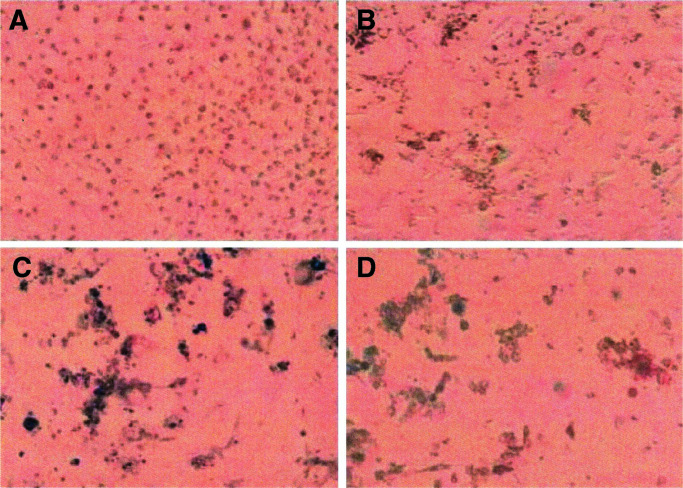
Pictures of malignant karyotyped cells from patient GBM2 kept in culture flasks after irradiation with ^137^Cs-*γ* radiation at an absorbed dose of 100 Gy. The pictures were taken at day 1 (**A**), 7 (**B**), 14 (**C**) and 20 (**D**) after irradiation.

In order to further evaluate if a sterilisation dose of 100 Gy was sufficient, one million cells were irradiated and plated in tissue culture flasks. The cells were kept in the flasks surveyed by photographing in an inverted phase-contrast microscope repeatedly during up to 3 or 4 weeks after irradiation. In all cases, the number of viable cells decreased with time. Photographs were taken at 1, 7, 14 and 20 days after irradiation with ^137^Cs-*γ* rays. In Figure 2

, pictures of a typical culture (GBM2, irradiated with an absorbed dose of 100 Gy) are displayed. The upper left picture (A) of Figure 2 clearly shows the confluent cells and their nuclei at day 1 after irradiation. The cells seem to be viable, although the proliferation is very limited since very few mitotic cells can be detected. The upper right picture (B) of Figure 2, taken at day 7, shows dark clusters of dead or dying cells and a few intact living cells are seen as well. Picture (C) in the lower left corner of Figure 2 that is taken at day 14 shows almost no viable cells and thus no proliferating cells. Dark clouds of dead cells dominate this picture. Finally, the lower right picture (D) of Figure 2, taken at day 20 after irradiation, shows no or very few cells that we can diagnose as viable among the dark clouds of dead cells.

## DISCUSSION

We have investigated the efficiency of the sterilisation of human malignant brain tumour cells in connection to a clinical study of ‘Brain ImmunoGene Tumour Therapy’ BRIGTT (Salford *et al*, 2002). In the present investigation, we studied nontransfected human glioma cells irradiated with ^137^Cs-*γ* rays at different absorbed doses (20, 40, 60, 80, 100 and 120 Gy).

The cell viability was studied by applying the MTT assay to cells irradiated at 20–120 Gy in 20 Gy intervals. The results of optical density measurements of the MTT assay were plotted as a function of absorbed dose and the data were fitted to an exponential model (equation (1)), which resulted in the parameters *δS*, *n* and mean lethal dose *D*_0_.

A constant fraction, *δS*, of ‘viable cells’ at high absorbed doses, was introduced to account for the fraction of cells still viable after an absorbed dose of 20 Gy or higher. The data for the extrapolation number at *D*=0 and mean lethal dose *D*_0_ obtained by fitting the MTT assay data are displayed in Table 2. Diagrams with both the constant fraction *δS* taken into account and with *δS* assumed to be zero are displayed in the figure. The parameters *δS*, extrapolation number *n* and mean lethal dose *D*_0_ obtained from the MTT assay were compared for cells from different patients. The mean values of the various parameters in the model with *δS* taken into account for the malignant karyotyped cells were determined to be *n*=0.85±0.06 and *D*_0_=12.4±3.2 Gy at *δS*=0.14±0.03. In case the value of *δS* was assumed to be zero, the corresponding mean values were determined to be *n*=0.86±0.16 and *D*_0_=30.0±8.1 Gy. The viability parameters of normal cells were found to be quite different between malignant karyotype cells and the cells of normal karyotype.

The cell proliferation was determined by the use of the ^3^H-thymidine incorporation assay. The count rate data, from measuring ^3^H in a scintillation counter, were plotted as a function of absorbed dose and then fitted to an exponential model (equation (1)). The values of the ^3^H-thymidine assay for the extrapolation number *n* and mean lethal dose *D*_0_ are displayed in Table 1, both with *δS* taken into account and with the value of *δS* assumed to be zero.

The parameters *δS*, extrapolation number *n* and mean lethal dose *D*_0_ obtained from the ^3^H proliferation study was compared for cells from different patients. In this comparison, we found no apparent difference between malignant karyotype cells and the cells of normal karyotype. In practice, this does not matter because there are no normal cells administered to the patient at the immunisation in order to avoid the risk of autoimmune reactions.

The mean values of the various parameters *n* and *D*_0_ and *δS* in the model for the malignant karyotyped GBM cells in this study were determined to be *n*=0.949±0.004, *D*_0_=5.8±0.6 Gy and *δS*=0.057±0.005. In the case where the value of *δS*=0, the values of the extrapolation number and the mean lethal dose were *n*=1.00±0.02 and *D*_0_=8.38±1.62 Gy, respectively.

Other previously documented studies of the intrinsic cellular radiation sensitivity of noninfected human glioblastoma cell lines have mostly used irradiation at absorbed dose levels below 12 Gy (Malaise *et al*, 1986; Taghian *et al*, 1992; Allam *et al*, 1993; Taghian *et al*, 1995; Wilkins *et al*, 1996; Budach *et al*, 1997; Raaphorst *et al* 1999). The first study of five human glioma cell lines resulted in the mean lethal dose *D*_0_=1.4±0.4 Gy (Malaise *et al*, 1986). A second study of 10 human glioblastoma cell lines *in vitro* resulted in the mean lethal dose *D*_0_=1.6±0.5 (Taghian *et al*, 1992, 1995) and a third study of 11 human glioblastoma cell lines *in vitro* resulted in a mean inactivation dose of *D*_0_=2.0±0.6 (Allam *et al*, 1993). Radiation survival curves for U373MG and U87MG human glioma cells, under the condition of high-dose rate up to 10 Gy resulted in the mean lethal dose values of 1.0 and 0.7 Gy, respectively (Wilkins *et al*, 1996; Raaphorst *et al* 1999). The results obtained in our study from three new GBM cell cultures yielded the mean lethal (nonproliferating) dose *D*_0_ in the order of 6–8 Gy. We have not found any radiosensitivity parameters in the literature at such a high absorbed dose as 100 Gy. In a recently published study, however, two human cell lines (GaMG and U-87MG) were irradiated up to 60 Gy. Their results indicate that irradiated malignant glioma subpopulations survive at this high absorbed dose, which is in agreement with our findings of a fraction of very radio-resistant cells (Gliemroth *et al*, 2003).

The experiments in the present investigation have been performed at absorbed doses up to 120 Gy. By using the *D*_0_, *n* and *δS* data for cell proliferation (^3^H-thymidine) and cell survival (MTT), it is possible to estimate the probability of proliferated and surviving cells, respectively, by equation (3).

**Figure 3:**
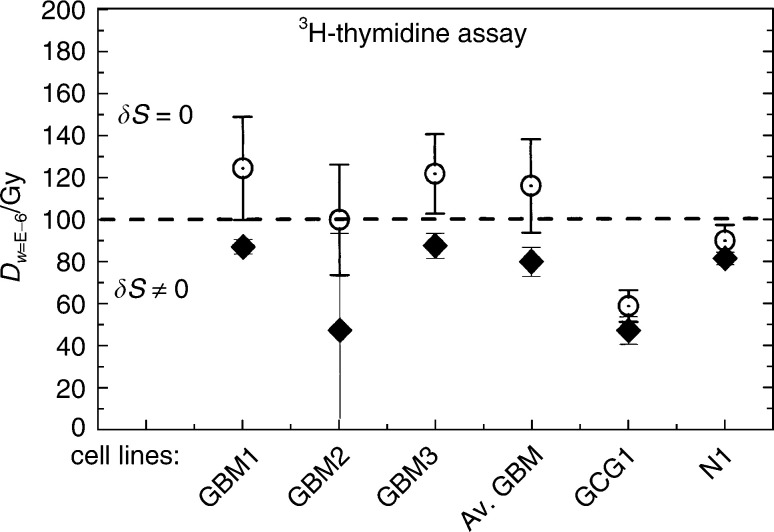
Adsorbed doses *D*_*w*=E−6_ calculated from survival ratios of ^3^H-thymidine incorporation assays corresponding to a probability *W*(*L*∣*V*)=10^−6^ (*w*=E−6) for cells still being viable after radiation. The average GBMs are calculated from patients GBM1, GBM2 and GBM3. The uncertainty bars represent 1 s.d. Keys to the abbreviations are given under the paragraph ‘Karyotyping’.

**Figure 4:**
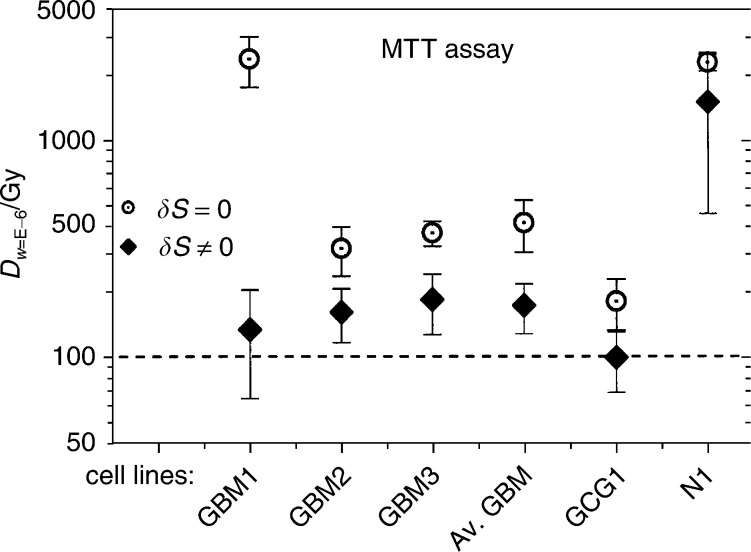
Adsorbed doses *D*_*w*=E−6_ calculated from survival fractions from the MTT assay corresponding to a probability *W*(*L*∣*V*)=10^−6^ (*w*=E−6) for cells still being viable after radiation. The average GMs are calculated from patients GBM1, GBM2 and GBM3. The uncertainty bars represent 1 s.d. Keys to the abbreviations are given under the paragraph ‘Karyotyping’.

The number of cells injected into the patient is about 2 × 10^6^ for each immunisation procedure. We want to estimate the absorbed dose for which there is only one single proliferating cell present, that is, when the proliferation probability is about 0.5 × 10^−6^≈10^−6^. The absorbed doses corresponding to the probability *W*(*L*∣*V*)=10^−6^ for the different cell lines are estimated from equation (5) and are displayed in Figure 3

from survival ratios of ^3^H-thymidine incorporation assays and in Figure 4

from survival fractions from the MTT assay.

Using the *n* and *D*_0_ data from the proliferation test given in Table 1, the absorbed dose *D*_*w*=E−6_ corresponding to one viable proliferating cell was calculated. From the results displayed in Figure 3, the absorbed dose *D*_*w*=E−6_ was estimated to be around 100 Gy. No significant difference was found in the absorbed dose *D*_*w*=E−6_ for the probability *W*(*L*∣*V*)=10^−6^ of cells not affected between the cases *δS*≠0 and =0 for cell proliferation (^3^H-thymidine).

Using the *n* and *D*_0_ data from the cell viability MTT assay in Table 2, the absorbed dose *D*_*w*=E−6_ was estimated to be around 200 Gy when using the data obtained at *δS*=0, but as high as 800 Gy when *δS*≠0. Thus, the cells seem to be viable up to very high doses. The most critical safety issue is, however, that the cells injected into the patient do not proliferate. Thus, a hypothetical absorbed dose around 100 Gy is, according to our findings, sufficient for reaching the negligible probability (10^−6^) for cell division.

In our studies of the plating efficiency, we observed a reduction in the total number of cells, which means that both cell division and survival are diminished. There is no visual difference in the observed cell survival pattern at day 20 in four cell cultures of malignant karyotype and one of normal karyotype. This observation confirms that we, *in vitro*, have apparently normal-looking cells 1 week after irradiation and that all the cells were dead and could not form colonies 4 weeks after irradiation, that is, 100 Gy is a safe dose for sterilisation with two million malignant brain tumour cells to be used for immunisation.

Thus based on the present investigation of the intrinsic cellular radiation sensitivity of nontransfected cells, we recommend an absorbed dose of 100 Gy for sterilisation of the 2 million cells used for immunisation of patients in the clinical study.

In the clinical study, the patients’ own tumour cells were infected with an adenovirus-expressing human IFN-gamma (100 MOI). The day after infection, the transfected cells were irradiated with 100 Gy. The sterilised cells administered to the patient were examined for the degree of expression of the IFN-*γ* gene by using ELISA measurements of IFN-*γ* production. The cells were plated 50 000–100 000 cells in 24-well plates and media were collected at different time points. The IFN-*γ* production of the irradiated cells after days 2, 3, 4 was typically in the order of 1–5 *μ*g ml^−1^ per million cells.

At the day after transfection, the immunisation of the patient takes place soon after the cells have been irradiated with 100 Gy. The irradiated cells cannot proliferate but they survive for some time in the skin of the patient, during which they produce their abnormal proteins and also the IFN-*γ*. This alerts the immune system and leads to a production of activated T-lymphocytes, which have the capability of passing through the blood–brain barrier. Out in the brain parenchyma, the activated T cells are free to seek actively for the tumour cells, both in the original tumour and in the surrounding brain with its migrating ‘guerrilla’ cells (Salford *et al*, 2002).
